# Effectiveness of HIV Risk Reduction Interventions among Men who have Sex with Men in China: A Systematic Review and Meta-Analysis

**DOI:** 10.1371/journal.pone.0072747

**Published:** 2013-08-30

**Authors:** Hongyan Lu, Yu Liu, Kapil Dahiya, Han-Zhu Qian, Wensheng Fan, Li Zhang, Juntao Ma, Yuhua Ruan, Yiming Shao, Sten H. Vermund, Lu Yin

**Affiliations:** 1 Institute for AIDS/STD Prevention & Control, Beijing Center for Disease Prevention and Control, Beijing, China; 2 Vanderbilt Institute for Global Health, Vanderbilt University School of Medicine, Nashville, Tennessee, United States of America; 3 Department of Public Health, College of Health and Human Services, Western Kentucky University, Bowling Green, Kentucky, United States of America; 4 Department of Medicine, Vanderbilt University School of Medicine, Nashville, Tennessee, United States of America; 5 Medical Library of Chinese People’s Liberation Army, Beijing, China; 6 State Key Laboratory for Infectious Disease Prevention and Control, and National Center for AIDS/STD Control and Prevention (NCAIDS), Chinese Center for Disease Control and Prevention (China CDC), Beijing, China; 7 Collaborative Innovation Center for Diagnosis and Treatment of Infectious Diseases, Hangzhou, China; 8 Department Pediatrics, Vanderbilt University School of Medicine, Nashville, Tennessee, United States of America; Alberta Provincial Laboratory for Public Health/University of Alberta, Canada

## Abstract

**Objective:**

To evaluate the effect of risk reduction interventions on HIV knowledge, attitudes and behaviors among men who have sex with men (MSM) in China.

**Methods:**

We performed a systematic review and meta-analysis of HIV risk reduction intervention studies among Chinese MSM. The summary difference of standardized mean differences (SMD) between both study arms or between pre- and post-intervention assessments were defined as the effect size (ES); ES was calculated using standard meta-analysis in random effects models.

**Results:**

Thirty-four eligible studies were included in the analysis, including two randomized clinical trials (RCT), six quasi-experimental studies, six pre-and-post intervention studies, and twenty serial cross-sectional intervention studies. These studies showed an increase in consistent condom use with any male sexual partners (mean ES, 0.46; 95% confidence interval [CI], 0.35–0.56), with regular sexual partners (mean ES, 0.41; 95% CI, 0.18–0.63), and casual sexual partners (mean ES, 0.52; 95% CI, 0.24–0.79). The analysis of ten studies that measured the impact on uptake of HIV testing also showed a positive result (mean ES, 0.55; 95% CI, 0.38–0.71). The risk reduction interventions also improved HIV/AIDS-related knowledge (mean ES, 0.77; 95% CI, 0.60–0.94) and attitudes (mean ES, 1.35; 95% CI, 0.91–1.79), but did not reduce prevalence of HIV (mean ES, 0.23; 95% CI, 0.02–0.45) and syphilis infections (mean ES, −0.01; 95% CI, −0.19–0.17). There was significant heterogeneity among these studies.

**Conclusions:**

On aggregate, HIV risk reduction interventions were effective in reducing risky behaviors and improving knowledge and attitudes among Chinese MSM, but were not associated with a change in the prevalence of HIV and syphilis. Future studies should use incidence as definitive study outcome.

## Introduction

Men who have sex with men (MSM) have become one of main subgroup populations at high risk of HIV infection in China in the past decade [1]. Chinese MSM tend to live in large- or middle-size cities rather than in small towns and rural areas for numerous considerations, e.g., there are more job opportunities; it is easier to find sexual partners; there are more socially tolerant environments which also keep some MSM away from their family members and acquaintances in their hometowns. MSM have lived in situations of repression, negative feedback, and discrimination and stigma. They often are poorly informed as to their sexual risks; a desire for intimacy and sexual fulfillment tends to outweigh the possible consequences of unprotected sex and the risks associated with it. Risky behaviors can occur in the context of an increasing number of MSM with HIV infection, some in acute stages of infection with very high HIV viral loads. The twin-epidemic of HIV and of STI can increase HIV viral expression and break down the integrity of mucosal surfaces and can further recruit HIV target cells to the infected area. HIV has spread quickly among Chinese MSM particularly in urban areas [2], [3], [4]; a national survey in 61 cities in years 2008 and 2009 showed 4.9% prevalence rate [5]. It is an urgent need to find effective intervention approaches to respond to the emerging epidemic among MSM in China as well as in other areas of the world [6].

Attitudes towards sex and sexual behaviors in China have evolved over thousands of years, but have advanced rapidly in recent years, reflecting cultural input consequent to industrialization and Western cultural norms and values in the past 30 years. Now people in China tend to be more tolerant towards homosexuality which has begun to be considered as a legitimate lifestyle choice. Although Chinese government has prioritized HIV prevention programs for MSM population, these programs often do not involve gay community and their community based organizations (CBOs), and therefore, their impact may be limited [7]. Unprotected sex and frequent change of sexual partners are prevalent among Chinese MSM, particularly among young MSM [8], and abuse of alcohol and club drugs further increase unprotected sex in a group of MSM [9], [10], [11] and put MSM at higher risk of contracting and transmitting HIV.

Two recently published meta-analyses evaluated the efficacy of HIV prevention intervention among MSM in China, suggesting that interventions may increase condom use, uptake of HIV testing, and HIV-related knowledge [12], [13]. There were numerous more recent intervention studies evaluating the efficacy on HIV-related behaviors, attitudes, and knowledge in China [14], [15], [16], [17], [18]. Hence, we conducted an updated systematic review and meta-analysis to evaluate the effects of HIV risk reduction interventions on knowledge, attitudes, behaviors and disease prevalence among Chinese MSM.

## Results

### Results from Literature Search

**Figure 1:**
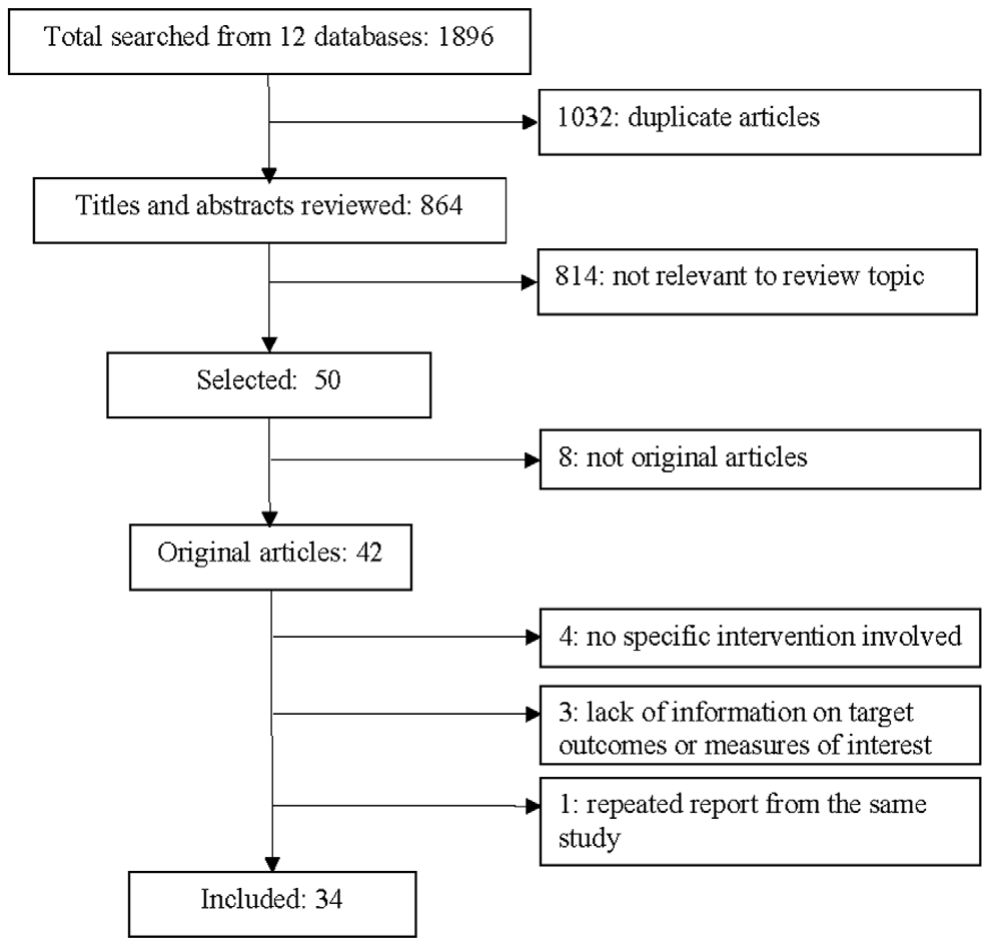
Flow diagram of the literature search process^1^. ^1^ Twelve databases included: 1) AMED; 2) BNI; 3) EMBASE; 4) CNKI; 5) CQVIP; 6) EconLit; 7) ERIC; 8) Medline; 9) PsycINFO; 10) Scopus; 11) ISI Web of Science; 12) Wanfang Data.

Our search yielded 1896 entries from twelve electronic databases (Figure 1); 864 titles and abstracts were reviewed and 1032 duplicates were removed. We excluded 814 citations because they did not meet one or more of the inclusion criteria. Out of 50 potential relevant papers for full text reviewing, 16 were further excluded because of not original article (i.e., editorial, comment, or review; k = 8), no specific intervention involved (k = 4), lack of information on target outcomes (k = 3), and repeated report from the same study (k = 1). The excluded articles are listed in Appendix S1. We included 34 studies in our systematic review.

Of 34 studies, study design included: two RCT [14], [19], six quasi-experimental studies [15], [17], [20], [21], [22], [23], six self-pre-and-post intervention studies without control groups [18], [24], [25], [26], [27], [28], and twenty serial cross-sectional studies (Table 1). A large variation of rigor scores was noted, ranging from 0 to 8, with a mean score of 2.5. One study had a rigor score of zero [29], nineteen had a score of one, and only five had a score of ≥6 [14], [19], [20], [22], [23] (Table 2).

**Table 1:** Characteristics of HIV intervention prevention studies among Chinese men who have sex with men.

Publication	City (trial period)	Study participants			Description of interventions		Study design	Follow-up (months)	Drop-out (%)
		Recruitment	No. of participants (age mean, age range)						
			Intervention	Comparison	Intervention	Comparison			
Gao et al. [20], 2005	Chengdu (N/A)	EBS, RDS	135→135^A^ 140→140^B^ (N/A, 16–46)	145→145 (N/A, 16–46)	A: self-facilitate peer-led intervention; B: social-facilitate peer-led intervention	No specific	QES	5	0
Song et al. [24], 2005	Shenzhen (N/A)	EBS	109→71 (24, 16–46)	N/A	Multi-way intervention	N/A	SPIS	0	35
Wang et al. [21], 2005	Chengdu (N/A)	RDS	20→150 (N/A, 16–42)	150→150 (N/A, 16–42)	Multi-way intervention	No specific	QES	5	14
Xu et al. [29], 2006	Chengdu & Kunming (2005)	EBS	48→48 (32, 18–69)	N/A	Multi-way intervention	N/A	SCIS	60	N/A
Gao et al. [22], 2007	Chengdu (N/A)	EBS, RDS	80→80 (25, 17–50)	80→80 (25, 17–50)	Peer-led intervention	No described	QES	5	0
Lau et al. [19], 2008	Hong Kong (N/A)	EBS, WDS	238→140 (N/A, 18–41+)	239→140 (N/A, 18–41+)	Internet-based intervention	Educational materials distributed	RCT	6	41
Liu et al. [58], 2008	Chongqing (2006–2007)	EBS	180→207 (23, N/A)	N/A	Multi-way intervention	N/A	SCIS	12	N/A
Wang et al. [33], 2008	Mianyang (2006–2007)	RDS	201→200 (24, 16–57)	N/A	Peer-led intervention	N/A	SCIS	6	N/A
Zhu et al. [25], 2008	Hefei, Wuhu & Fuyang (N/A)	PDR	218→170 (24, 18–61)	N/A	Peer-led intervention	N/A	SPIS	3	22
Cao et al. [59], 2009	Shenyang, Chengdu & Nanjing (2007)	EBS	484→553 (21, 16–45)	N/A	Multi-way intervention	N/A	SCIS	6	N/A
Feng et al. [60], 2009	Chongqing (2006–2007)	RDS	1000→772 (28, ≥18)	N/A	Multi-way intervention	N/A	SCIS	12	N/A
Wang M et al. [61], 2009	Wuhan (2006)	SBS	222→224 (N/A, 15–24)	N/A	Multi-way intervention	N/A	SCIS	6	N/A
Wang Y et al. [62], 2009	Mianyang (2006–2008)	RDS	201→200→203 (24, 16–57)	N/A	Peer-led intervention	N/A	SCIS	24	N/A
Xiu et al. [63], 2009	Qingdao (2007–2008)	EBS	216→199 (27, 18–50)	N/A	Multi-way intervention	N/A	SCIS	N/A	N/A
Xu et al. [32], 2009	Wuhan (2007–2008)	EBS	253→154 (27, 15–61)	N/A	Multi-way intervention	N/A	SCIS	6	N/A
Zeng et al. [64], 2009	18 cities (2006–2008)	RDS, WDS	5178→5460 (26, ≥18)	N/A	Multi-way intervention	N/A	SCIS	24	N/A
Zhang et al. [23], 2009	Mianyang & Yibin (2007)	PDS	200→200 (N/A, 18–35+)	200→200 (N/A, 18–35+)	Peer-led intervention	Routine HIV intervention	QES	6	0
Ding et al. [34], 2010	Chongqing (2006–2008)	SBS	1000→1044→743 (27, 18–68)	N/A	Multi-way intervention	N/A	SCIS	24	N/A
He et al. [26], 2010	Wuhu (2006–2008)	RDS	360→306 (23, 15–48)	N/A	Multi-way intervention	N/A	SPIS	24	15
Ma et al. [65], 2010	Xiamen (2008–2009)	RDS	98→140→154 (25, N/A)	N/A	Multi-way intervention	N/A	SCIS	14	N/A
Meng et al. [27], 2010	Pulan (2009)	SBS	62→62 (28, 19–49)	N/A	Peer-led intervention	N/A	SPIS	10	0
Nong et al. [66], 2010	Nanning (2007–2008)	EBS	230→452→452 (25, N/A)	N/A	Multi-way intervention	N/A	SCIS	12	N/A
Zhang et al. [30], 2010	Guilin (2008–2009)	EBS	315→346 (28, 18–51+)	N/A	Multi-way intervention	N/A	SCIS	12	N/A
Li et al. [35], 2011	Nanjing (2008–2010)	SBS	606→616→400 (28, ≥18)	N/A	Multi-way intervention	N/A	SCIS	24	N/A
Qu et al. [67], 2011	Hohhot & Baotou (2008–2009)	SBS	706→767 (27, 18–63)	N/A	Peer-led intervention	N/A	SCIS	12	N/A
Wang F et al. [68], 2011	Yingtan (2009–2010)	PDS	135→134 (27, N/A)	N/A	Multi-way intervention	N/A	SCIS	12	N/A
Wang L et al. [69], 2011	N/A (2008–2010)	SBS	500→496 (23, ≥18)	N/A	HIV testing intervention	N/A	SCIS	24	N/A
Wang Y et al. [28], 2011	Nanchang (2006–2007)	EBS	101→101 (N/A, N/A)	N/A	Peer-led intervention	N/A	SPIS	12	0
Wu et al. [31], 2011	Wuhu (2009–2010)	SBS	244→179 (20, 18–25)	N/A	Multi-way intervention	N/A	SCIS	6	N/A
Hao et al. [14], 2012	Nanjing (2008–2009)	RDS	149→100 (28, 18–73)	146→111 (28, 18–73)	Enhanced voluntary counseling	Standard voluntary counseling	RCT	6	28
Tan et al. [15], 2012	Shenzhen (2009–2010)	EBS	111→120 (28, N/A)	105→98 (26, N/A)	IEC intervention	Educational materials distributed	QES	12	N/A
Wang et al. [16], 2012	Harbin (2006–2010)	SBS	400→419→451→450→413 (N/A, 18–79)	N/A	Multi-way intervention	N/A	SCIS	48	N/A
Duan et al. [17], 2013	Mianyang & Yibin (2006–2008)	PDS	200→200 (N/A, ≥18)	200→200 (N/A, ≥18)	Peer-led intervention	Routine HIV intervention	QES	12	N/A
Guo et al. [18], 2013	Langfang (2007)	WDS, PDS, EBS	233→200 (N/A, ≥18)	N/A	HIV testing intervention	N/A	SPIS	3	14

**NOTE:** EBS: establishment-based sampling; RDS: respondent-driven sampling; PDS: peer-driven sampling; WDS: web-driven sampling; SBS: snowball sampling; IEC: information, education, communication; QES: quasi-experimental study, SCIS: serial cross-sectional intervention studies; SPIS: self-pre-and-post intervention studies without comparison group; RCT: randomized control trial; N/A: Not available.

**Table 2:** Quality assessment of study design (rigor score*).

Publication	Cohort (a)	With control group (b)	Pre/post intervention (c)	Random assignment (d)	Random selection for assessment (e)	Sample size >100 (f)	Follow-up≥80% (g)	Comparable socio-demographics between study arms (h)	Comparable outcome measures at baseline (i)	Total
Gao et al. [20], 2005	1	1	1	0	0	1	1	0.5	1	6.5
Song et al. [24], 2005	1	0	1	0	0	1	0	0	0	3
Wang et al. [21], 2005	1	1	1	0	0	1	1	0.5	0	5.5
Xu et al. [29], 2006	0	0	0	0	0	0	0	0	0	0
Gao et al. [22], 2007	1	1	1	0	0	1	1	1	1	7
Lau et al. [19], 2008	1	1	1	1	1	1	0	1	1	8
Liu et al. [58], 2008	0	0	0	0	0	1	0	0	0	1
Wang et al. [33], 2008	0	0	0	0	0	1	0	0	0	1
Zhu et al. [25], 2008	1	0	1	0	0	1	0	0	0	3
Cao et al. [59], 2009	0	0	0	0	0	1	0	0	0	1
Feng et al. [60], 2009	0	0	0	0	0	1	0	0	0	1
Wang M et al. [61], 2009	0	0	0	0	0	1	0	0	0	1
Wang Y et al. [62], 2009	0	0	0	0	0	1	0	0	0	1
Xiu et al. [63], 2009	0	0	0	0	0	1	0	0	0	1
Xu et al. [32], 2009	0	0	0	0	0	1	0	0	0	1
Zeng et al. [64], 2009	0	0	0	0	0	1	0	0	0	1
Zhang et al. [23], 2009	1	1	1	0	0	1	1	0	1	6
Ding et al. [34], 2010	0	0	0	0	0	1	0	0	0	1
He et al. [26], 2010	1	0	1	0	0	1	0	0	0	3
Ma et al. [65], 2010	0	0	0	0	0	1	0	0	0	1
Meng et al. [27], 2010	1	0	1	0	0	0	1	0	0	3
Nong et al. [66], 2010	0	0	0	0	0	1	0	0	0	1
Zhang et al. [30], 2010	0	0	0	0	0	1	0	0	0	1
Li et al. [35], 2011	0	0	0	0	0	1	0	0	0	1
Qu et al. [67], 2011	0	0	0	0	0	1	0	0	0	1
Wang F et al. [68], 2011	0	0	0	0	0	1	0	0	0	1
Wang L et al. [69], 2011	0	0	0	0	0	1	0	0	0	1
Wang Y et al. [28], 2011	1	0	1	0	0	1	1	0	0	4
Wu et al. [31], 2011	0	0	0	0	0	1	0	0	0	1
Hao et al. [14], 2012	1	1	1	1	1	1	0	1	1	8
Tan et al. [15], 2012	0	1	0	0	0	1	0	1	1	4
Wang et al. [16], 2012	0	0	0	0	0	1	0	0	0	1
Duan et al. [17], 2013	0	1	0	0	0	1	0	0	0	2
Guo et al. [18], 2013	1	0	1	0	0	1	1	0	0	4

* One point score for meeting each of the following items (if data were not available in the articles for any item, 0.5 was recorded):

(a) was a prospective cohort,

(b) used a comparison arm,

(c) collected pre and post intervention data,

(d) used random assignment of participants to study arms,

(e) did random sampling for assessments,

(f) sample size >100,

(g) follow-up rate ≥80%,

(h) had a comparison group with comparable socio-demographics such as age, education, race, employment, income, marital status and others [score “1” if >50% variables were comparable between study arms, and ‘0’ if not], and

(i) had a comparison arm with comparable outcome measures at baseline between study arms.

### Consistent Condom Use

**Figure 2:**
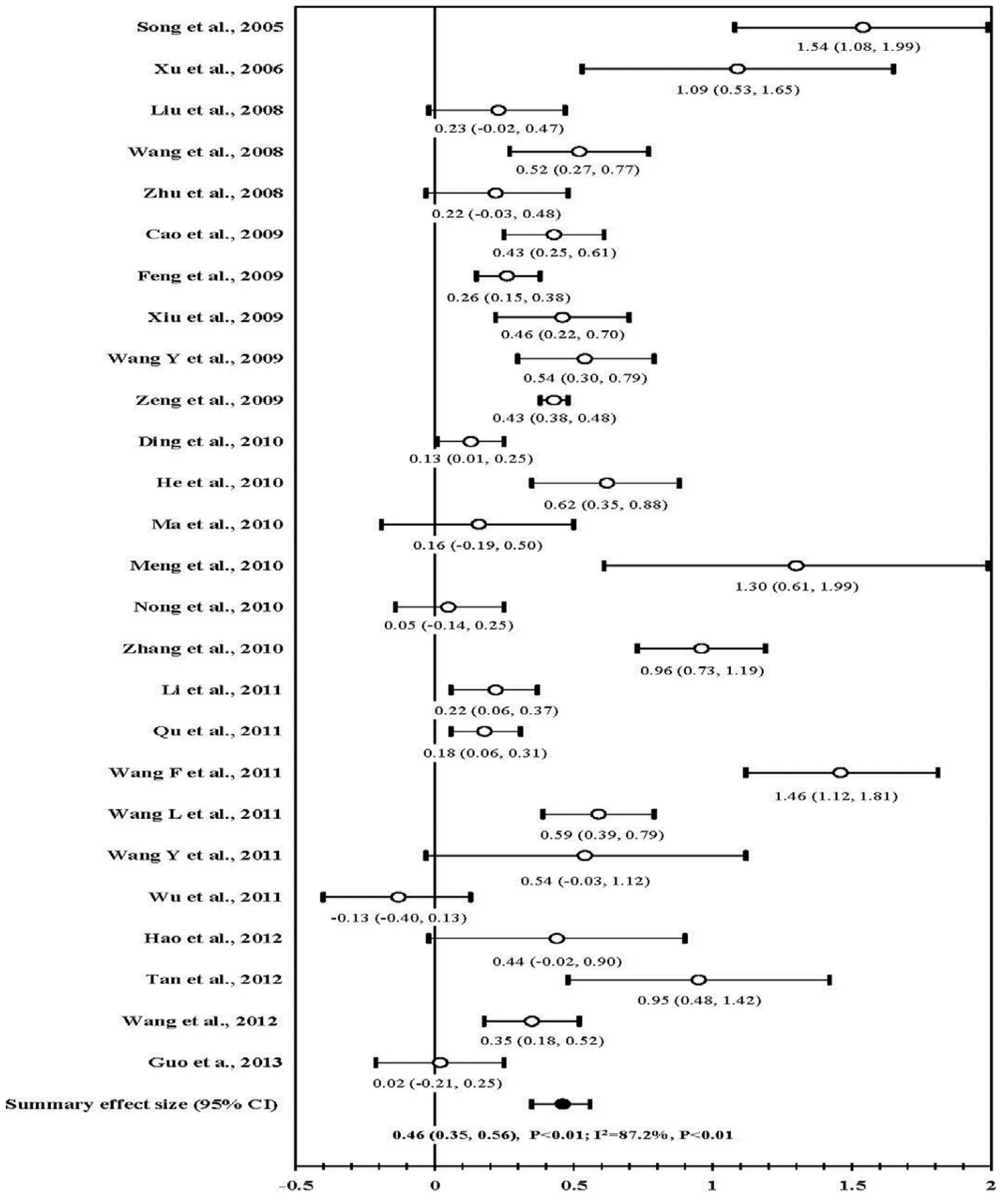
Forest plot of effect size: the impact of behavioral interventions on consistent condom use during anal intercourses with any male sexual partners among MSM in China.

A variety of condom use outcomes were reported, e.g., in anal sex with regular, casual, and/or mixed sexual partners and were measured during various recall periods, e.g., during last sexual encounter, in the past month, and/or the past six months (Table 3). The overall effectiveness of risk reduction interventions on consistent condom use with any sexual partners during anal intercourse is presented in Figure 2. Twenty five studies reported a positive association between interventions and consistent condom use, and 17 had statistical significance. Meta-analysis of these 25 studies showed that risk reduction intervention increased consistent condom use (mean ES: 0.46; 95% CI: 0.35, 0.56; *P*<0.01). Large heterogeneity was observed among these studies (I^2^ = 87.2%; *P*<0.01). The funnel plot show significant evidence of publication bias (Kendall tau = 0.31; *P* = 0.03; Egger’s t value = 3.86; *P*<0.01).

**Table 3:** Behavioral, biomedical, and knowledge outcomes of HIV intervention studies among Chinese MSM.

Publication	Consistent condom use (%)		Uptake of HIV testing (%)		HIV/AIDS-related knowledge and attitudes (%)		HIV/STI prevalence (%)	
	IG	CG	IG	CG	IG	CG	IG	CG
Gao et al. [20], 2005*	AI with CP^PM6^ 4.6→66.6 3.7→51.1 AI with RP^PM6^ 4.1→46.8 4.1→26.5	AI with CP^PM6^ 3.8→4.5 AI with RP^PM6^ 3.4→4.2	N/A	N/A	Knowledge 11.1→76.3 10.7→58.6 Attitude 16.3→67.4 14.2→61.4	Knowledge 11.0→11.7 Attitude 15.1→16.5	N/A	N/A
Song et al. [24], 2005	AI^PM6^ 11.9→63.0	N/A	N/A	N/A	Knowledge 60.7→84.2	N/A	N/A	N/A
Wang et al. [21], 2005	AI with RP 10.5→35.5 AI with CP 6.5→49.7	AI with RP 6.8→6.2 AI with CP 3.1→5.3	N/A	N/A	Knowledge 31.0→71.8 Attitude 34.1→68.5	Knowledge 32.3→32.5 Attitude 32.4→31.2	N/A	N/A
Xu et al. [29], 2006	AI^PM1^18.8→58.3	N/A	N/A	N/A	Knowledge 16.7→90.8	N/A	N/A	N/A
Gao et al. [22], 2007	AI with CP 4.3→76.8 AI with RP 3.1→46.2	AI with CP 2.9→4.2 AI with RP 3.2→4.8	N/A	N/A	Knowledge 11.3→86.3 Attitude 16.3→87.5	Knowledge 10.8→11.2 Attitude 15.0→16.2	N/A	N/A
Lau et al. [19], 2008	AI with CP^PM6^ 63.0→60.0 AI with RP^PM6^ 37.3→42.9	AI with CP^PM6^ 49.1→61.1 AI with RP^PM6^ 30.8→39.3	VCT^PM6^ 20.7→15.7	VCT^PM6^ 10.7→12.1	Knowledge 88.6→93.6	Knowledge 87.9→94.3	STD^PM6^ 5.7→2.9	STD^PM6^ 2.1→4.3
Liu et al. [58], 2008	AI^PM6^ 40.9→50.2 AI^PM0^ 64.3→75.8	N/A	18.3→58.0	N/A	Knowledge 35.6→65.2	N/A	N/A	N/A
Wang et al. [33], 2008	AI^PM6^ 31.5→52.0	N/A	26.9→45.5	N/A	Knowledge 68.6→76.6	N/A	N/A	N/A
Zhu et al. [25], 2008	AI^PM0^ 56.4→65.2 AI with CP^PM0^ 46.2→52.5 AI with RP^PM0^ 48.4→60.9	N/A	N/A	N/A	Knowledge** 14.71(2.59) →16.95(1.81)	N/A	N/A	N/A
Cao et al. [59], 2009	IAI^PM3^ 71.7→83.7 IAI^PM0^ 86.5→90.4	N/A	69.6→70.5	N/A	Knowledge 62.7→91.9	N/A	N/A	N/A
Feng et al. [60], 2009	AI^PM0&PM6^ 56.4→65.5 31.8→41.9	N/A	N/A	N/A	Knowledge 74.3→82.4	N/A	Syphilis 9.3→7.3 HIV 10.4→10.8	N/A
Wang M et al. [61], 2009	AI with CP^PM1^ 25.2→25.6 AI with RP^PM1^ 15.3→23.4	N/A	N/A	N/A	N/A	N/A	N/A	N/A
Wang Y et al. [62], 2009	AI^PM6^ 31.5→41.3→52.9 AI with CP^PM6^ 30.3→47.1→57.8 AI with RP^PM6^ 23.7→38.7→43.0 AI^PM0^ 54.0→73.1→82.8 AI with CP^PM0^ 65.2→74.2→88.9 AI with RP^PM0^ 51.7→71.1→82.0	N/A	N/A	N/A	N/A	N/A	N/A	N/A
Xiu et al. [63], 2009	AI^PM6^ 45.3→63.8	N/A	N/A	N/A	N/A	N/A	N/A	N/A
Xu et al. [32], 2009	AI with CP^PM6^ 40.3→39.0 AI with CP^PM0^ 50.2→70.6 AI with RP^PM6^ 38.3→48.7 AI with RP^PM0^ 37.7→56.6	N/A	37.9→36.4	N/A	N/A	N/A	N/A	N/A
Zeng et al. [64], 2009	AI^PM0&PM6^ 58.0→76.7 28.2→44.5	N/A	18.8→39.1	N/A	Knowledge 76.0→90.5	N/A	HIV 2.3→5.0	N/A
Zhang et al. [23], 2009	AI with CP^PM0^ 80.5→89.0 AI with RP^PM0^ 67.0→72.0	AI with CP^PM0^ 82.5→81.0 AI with RP^PM0^ 70.5→75.0	9.0→22.0	24.5→24.0	N/A	N/A	N/A	N/A
Ding et al. [34], 2010	AI^PM6^ 31.8→36.2→36.7 AI^PM0^ 56.4→61.2→64.4	N/A	18.9→35.2→32.0	N/A	Knowledge 90.0→89.5→90.7	N/A	HIV 10.4→12.5→17.0 Syphilis 9.3→8.5→8.5	N/A
He et al. [26], 2010	AI 9.7→22.9^PM6^ 13.3→25.5^PM1^ 14.7→41.2^PM0^	N/A	N/A	N/A	N/A	N/A	N/A	N/A
Ma et al. [65], 2010	AI^PM0^ 71.7→66.7→76.6	N/A	32.7→42.1→59.1	N/A	Knowledge 43.9→45.9→59.7	N/A	HIV 2.0→1.4→2.6 Syphilis 9.2→4.3→10.4 HCV 1.0→0.7→0.7	N/A
Meng et al. [27], 2010	AI^PM1^ 6.45→37.1 AI^PM0^ 27.4→77.4	N/A	N/A	N/A	N/A	N/A	N/A	N/A
Nong et al. [66], 2010	AI^PM6^ 43.0→34.6→45. 2 AI^PM0^ 72.2→64.4→75.1	N/A	28.3→38.7→44.7	N/A	N/A	N/A	HIV 0.87→1.99→2.43 Syphilis 10.43→8.18→11.28	N/A
Zhang et al. [30], 2010	AI^PM6^ 15.1→46.4	N/A	17.8→55.2	N/A	Knowledge 30.2→37.9	N/A	HIV 1.59→2.02	N/A
Li et al. [35], 2011	AI^PM6^ 41.9→56.8→50.8 AI^PM0^ 57.8→67.5→64.0 AI with CP^PM6^ 64.7→36.4→65.8 AI with RP^PM6^ 47.5→45.6→40.1	N/A	N/A	N/A	Knowledge 97.0→97.4→98.8	N/A	HIV 4.0→2.1→2.8 Syphilis 11.4→7.5→7.8	N/A
Qu et al. [67], 2011	AI^PM6^ 38.0→45.3 AI^PM0^ 81.4→82.5	N/A	N/A	N/A	Knowledge 70.7→81.7	N/A	HIV 1.7→1.7	N/A
Wang F et al. [68], 2011	AI^PM6^ 23.7→77.6 AI^PM0^ 49.6→89.6	N/A	N/A	N/A	Knowledge 63.0→95.5	N/A	N/A	N/A
Wang L et al. [69], 2011	AI^PM6^ 12.5→27.5 AI^PM0^ 58.9→75.8	N/A	44.4→65.2	N/A	Knowledge 56.8→87.0	N/A	HIV 6.2→5.6	N/A
Wang Y et al. [28], 2011	AI 84.8→93.2	N/A	N/A	N/A	Knowledge 81.1→88.2	N/A	N/A	N/A
Wu et al. [31], 2011	AI^PM6^ 29.4→25.0 AI^PM0^ 56.5→67.9	N/A	N/A	N/A	Knowledge 81.8→92.0	N/A	N/A	N/A
Hao et al. [14], 2012	AI^PM6^28.1→51.6 AI with CP^PM6^ 48.9→63.2 AI with RP^PM6^ 22.2→47.8	AI^PM6^ 27.4→33.3 AI with CP^PM6^ 46.7→47.5 AI with RP^PM6^ 21.4→31.1	N/A	N/A	N/A	N/A	N/A	N/A
Tan et al. [15], 2012	AI^PM6^ 39.2→61.6 AI^PM0^ 73.0→85.0	AI^PM6^ 42.9→→33.3 AI^PM0^ 64.0→65.3	69.4→90.8	52.4→56.1	Knowledge 73.0→91.7	Knowledge 66.7→68.4	N/A	N/A
Wang et al. [16], 2012	AI^PM6^ 38.7→36.3→ 41.8→44.1→52.9	N/A	26.2→31.0→ 37.0→56.4→47.2	N/A	N/A	N/A	HIV 1.0→2.9→ 3.5→5.1→7.5 Syphilis 9.2→15.5→ 14.4→22.4→15.7	N/A
Duan et al. [17], 2013	AI with CP^PM6^ 32.1→58.4 AI with RP^PM6^ 23.7→42.5 AI with CP^PM0^ 5.7→18.2 AI with RP^PM0^51.7→78.0	AI with CP^PM6^ 36.4→30.2 AI with RP^PM6^ 30.3→21.6 AI with CP^PM0^ 18.1→5.8 AI with RP^PM0^ 58.5→41.1	N/A	N/A	Knowledge** 15.1(2.3) →12.4(3.3)	Knowledge** 9.9(3.0) →11.6(3.6)	N/A	N/A
Guo et al. [18], 2013	AI^PM3^50.7→51.5	N/A	N/A	N/A	N/A	N/A	N/A	N/A

**NOTE:** MSM: men who have sex with men; IG: intervention group; CG: comparison group; AI: anal intercourse; CP: casual partners; RP: regular partners; VCT: voluntary counseling and testing;

* Gao et al. [20], 2005 conducted 2 independent intervention patterns with the same control group, including self-facilitate peer-led intervention and social-facilitate peer-led intervention;

** Knowledge scores as score mean (standard deviance).

The effectiveness was also shown in subgroup analyses by: (1) type of sexual partners (2) recall period, (3) number of study sites, (4) venue of recruiting participants, (5) type of risk reduction intervention, (6) study design, (7) sample size at baseline, and (8) rigor score (Table 4). In standardized deleted residual analysis, four studies [24], [27], [30], [31] were identified as outliers (standardized deleted residual = 3.40 [24], 2.02 [27], 2.05 [30],−2.23 [31]). Further sensitivity analyses were used to evaluate the stability of summary effect size in the meta-analysis by excluding the outlier studies. Summary effect sizes were not changed after these exclusions (Table 4).

**Table 4:** Subgroup and sensitivity analyses of consistent condom use with any sexual partners during anal intercourse.

Subgroup	No. of studies (k)	Combined ES (95% CI)	*P*-value	Heterogeneity	
				I^2^	*P*-value
Recall period on consistent condom use (months)					
Last sex	16	0.42 (0.28, 0.56)	<0.01	89.6%	<0.01
Past 6 months	19	0.48 (0.35, 0.60)	<0.01	89.5%	<0.01
Number of study sites					
One	20	0.51 (0.34, 0.67)	<0.01	88.8%	<0.01
Multiple	6	0.35 (0.22, 0.48)	<0.01	80.3%	<0.01
Venue of recruiting participants					
Establishment-based	10	0.59 (0.32, 0.85)	<0.01	89.2%	<0.01
Other	16	0.40 (0.28, 0.52)	<0.01	86.4%	0.01
Type of risk reduction interventions					
Peer-led	6	0.44 (0.22, 0.67)	<0.01	73.9%	0.01
Multi-way	20	0046 (0.33, 0.59)	<0.01	89.1%	<0.01
Study design					
Randomized clinical evaluation	1	0.44 (−0.02, 0.90)	–	–	–
Quasi-experimental evaluation	1	0.95 (0.48, 1.42)	–	–	–
Self-pre-and-post intervention evaluation	6	0.66 (0.23, 1.09)	<0.01	89.0%	<0.01
Serial cross-sectional evaluation	18	0.40 (0.29, 0.52)	<0.01	88.1%	<0.01
Sample size at baseline					
≤300	17	0.55 (0.34, 0.76)	<0.01	87.5%	<0.01
>300	9	0.38 (0.26, 0.50)	<0.01	88.2%	<0.01
Rigor score					
1	18	0.40 (0.29, 0.52)	<0.01	88.1%	<0.01
>1	8	0.66 (0.32, 1.01)	<0.01	86.1%	<0.01
**Sensitivity analyses**					
Song et al. [24], 2005 excluded	25	0.42 (0.32, 0.52)	<0.01	85.9%	<0.01
Meng et al. [27], 2010 excluded	25	0.44 (0.33, 0.55)	<0.01	87.3%	<0.01
Zhang et al. [30], 2010 excluded	25	0.43 (0.32, 0.53)	<0.01	85.8%	<0.01
Wu et al. [31], 2011 excluded	25	0.48 (0.37, 0.58)	<0.01	86.7%	<0.01

**NOTE:** ES: effect size; CI: confidence interval.

**Figure 3:**
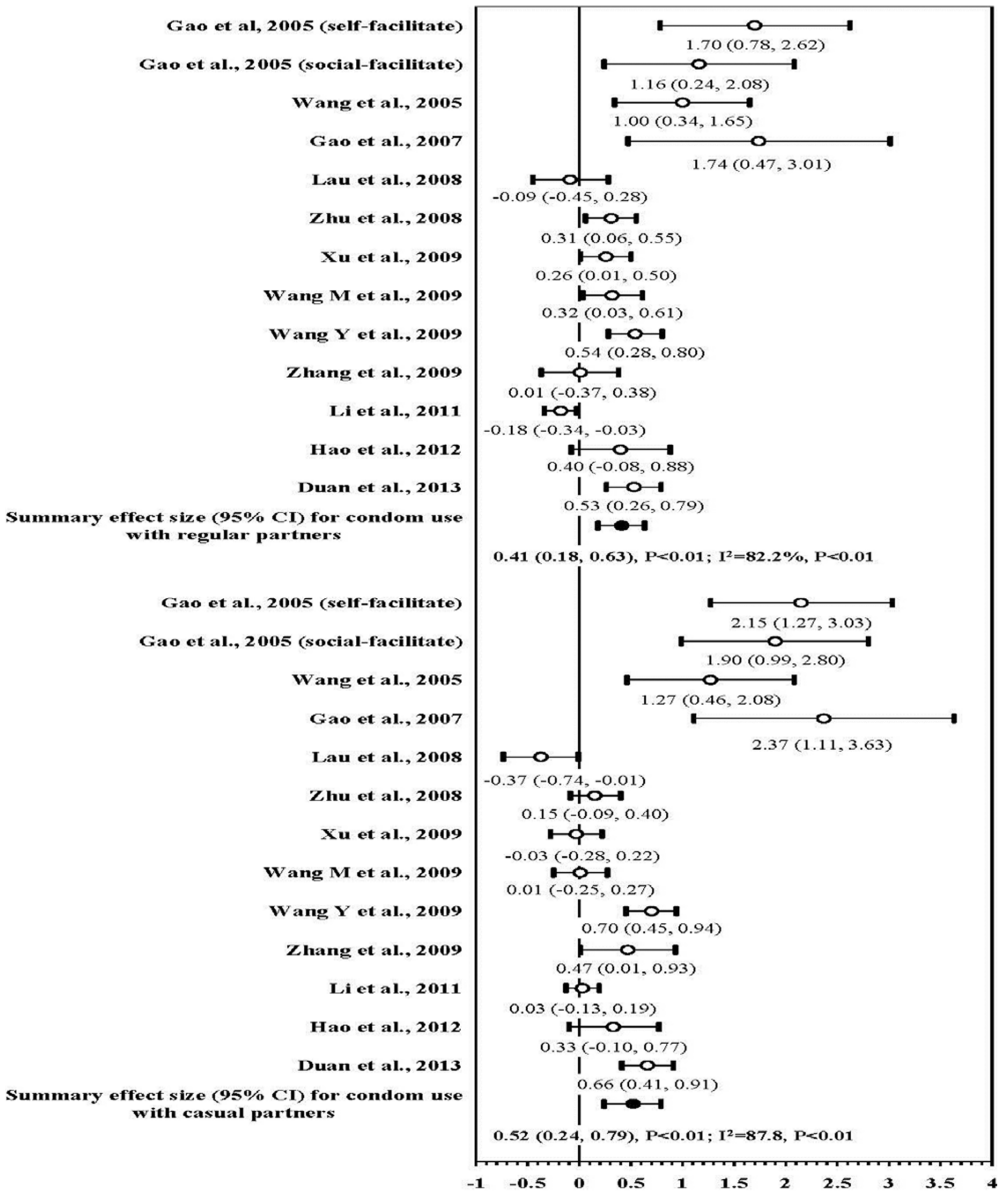
Forest plot of effect size: the impact of behavioral interventions on consistent condom use during anal intercourses among MSM in China by types of sexual partners.

Six studies presented separately the proportions of consistent condom use with regular and casual sexual partners during anal intercourse. The effect sizes in the meta-analysis were similar with regular sexual partners (mean ES, 0.41; 95% CI, 0.18–0.63), and with casual sexual partners (mean ES, 0.52; 95% CI, 0.24–0.79; Figure 3).

### Uptake of HIV Testing

**Figure 4:**
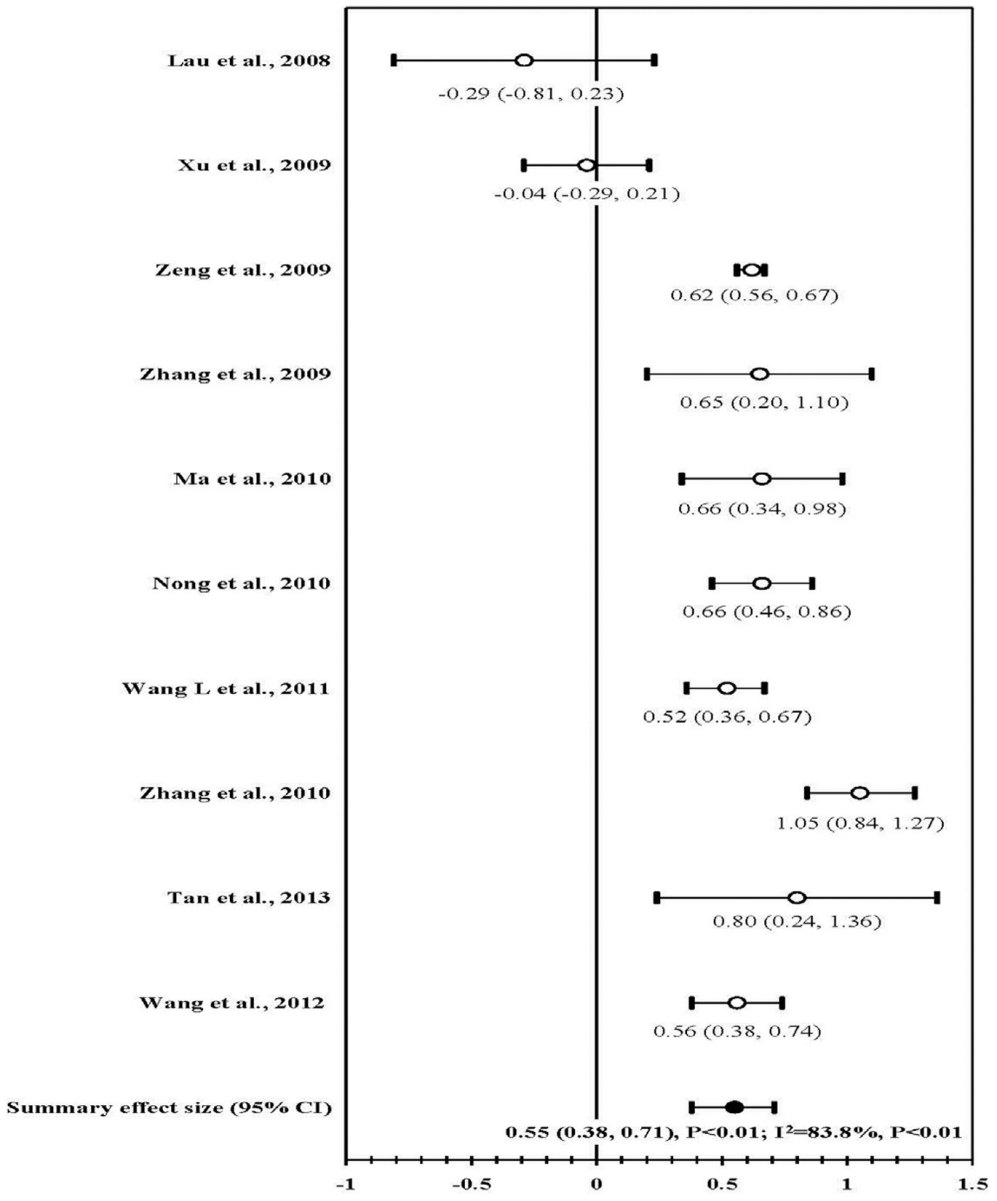
Forest plot of effect size: the impact of behavioral interventions on uptake of HIV testing among MSM in China.

Of ten studies evaluating uptake of HIV testing eight reported an increased proportion of taking HIV testing while two did not [19], [32]. Meta-analysis of ten studies showed a marked increase in taking HIV testing post intervention (mean ES, 0.55; 95% CI, 0.38–0.71; *P*<0.01). Substantial heterogeneity was found across these six studies (I^2^ = 83.8%, *P*<0.01; Figure 4).

### HIV/AIDS-related Knowledge and Attitudes

**Figure 5:**
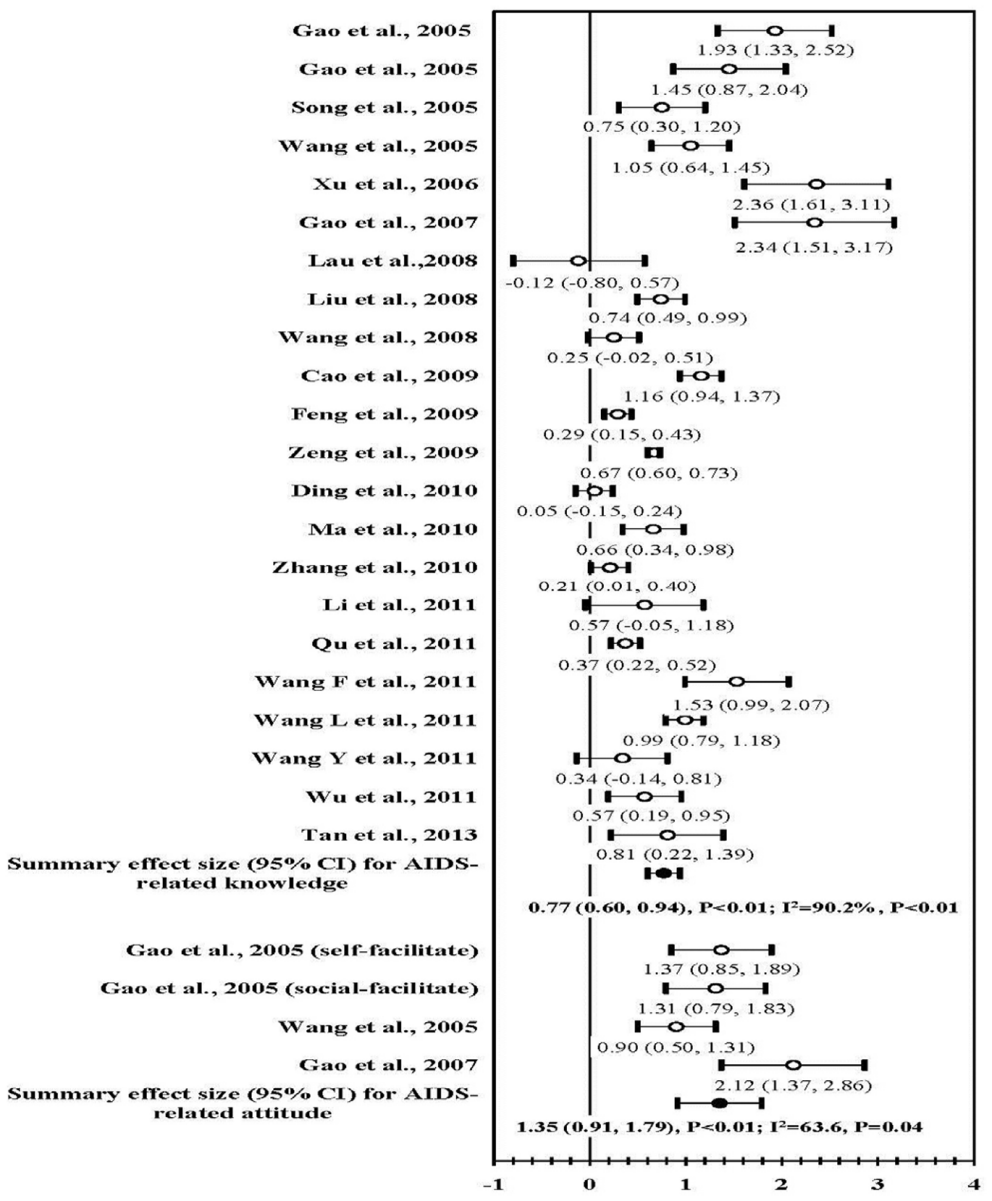
Forest plot of effect size: the impact of behavioral interventions on HIV/AIDS-related knowledge and attitude among MSM in China.

Of 21 studies reported HIV/AIDS knowledge outcome, 20 showed statistically significant increase, while 4 reported no statistically different change [28], [33], [34], [35] (Figure 5). Meta-analysis found a significant positive effect size (mean ES, 0.77; 95% CI, 0.60–0.94; *P*<0.01). Large statistical heterogeneity was observed (I^2^ = 90.2%; *P*<0.01).

Only three studies evaluated AIDS-related attitudes [20], [21], [22], and all found improvement of AIDS-related attitudes. The summary ES was 1.35 (95% CI, 0.91, 1.79; *P*<0.01), but large heterogeneity was noted (I^2^ = 63.6%, *P* = 0.04).

### HIV and Syphilis Infections

**Figure 6:**
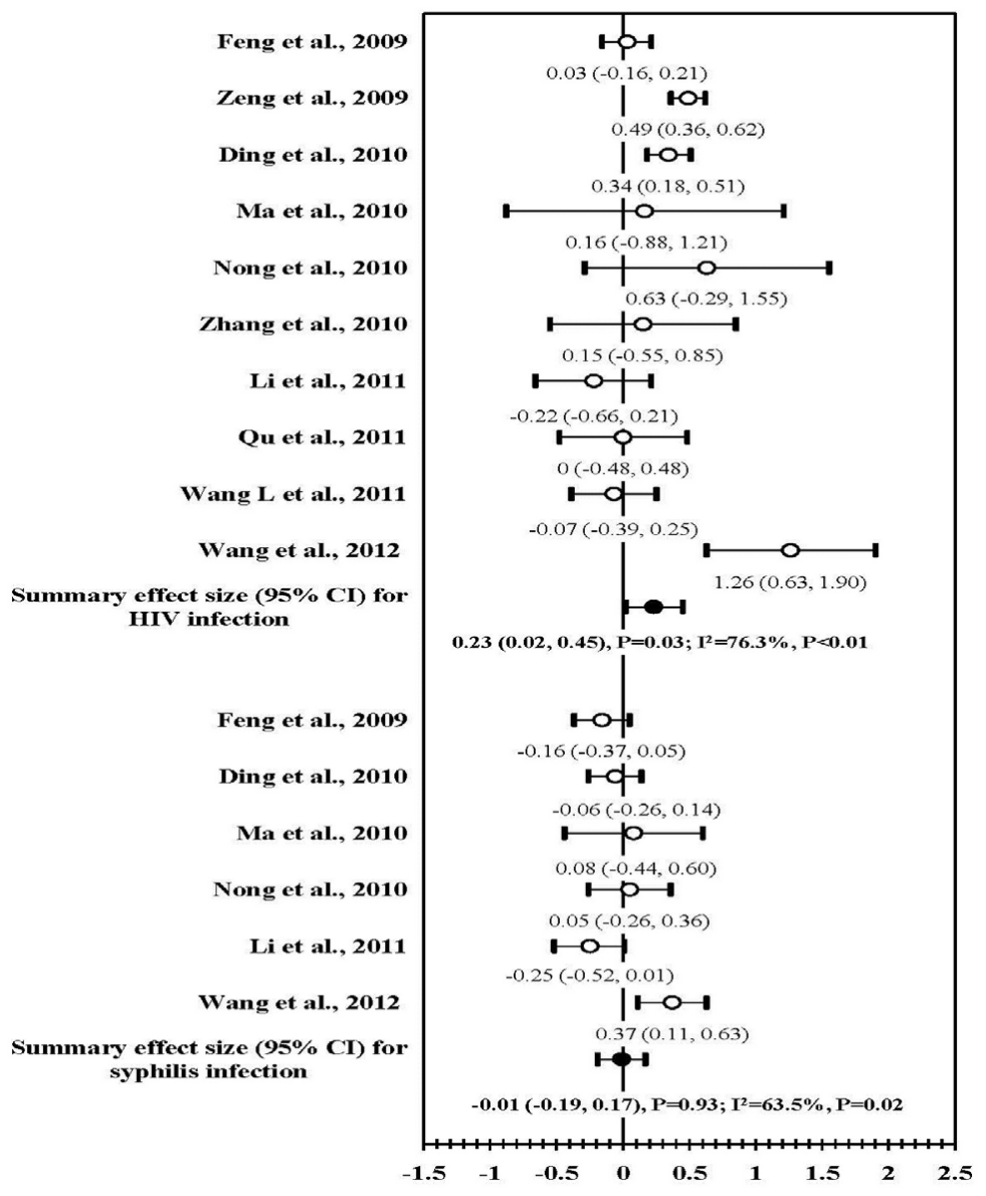
Forest plot of effect size: the impact of behavioral interventions on HIV and syphilis prevalence among MSM in China.

Ten serial cross-sectional studies assessed HIV/STI outcomes. Six studies had no summary effect on syphilis prevalence (mean ES, −0.01; 95% CI, −0.19, 0.17; *P* = 0.93), and ten studies had a positive overall effect on increasing HIV prevalence (mean ES, 0.23; 95% CI, 0.02, 0.45; *P* = 0.03). Large statistical heterogeneity was observed for two outcomes (Figure 6).

## Discussion

Our systematic review and meta-analysis evaluate the effectiveness of behavioral interventions on the HIV-related behaviors, knowledge and attitudes, as well as prevalence of HIV/STI among MSM in China. Compared to two previous meta-analytic reviews involving 16 [12] and 22 [13] individual studies in China, respectively, our review included 34 studies with four intervention study designs. Our meta-analysis confirmed previous reviews on increasing consistent condom use, HIV/AIDS knowledge [13], and uptake of HIV testing [12], [13]. Our review also evaluated the effect on HIV and syphilis prevalence, but showed no positive effect.

Consistent condom use is seen as the most relevant HIV-related behavior to evaluate effectiveness of interventions among MSM. Our meta-analysis found that a variety of behavioral interventions conducted in China were associated with a significant increase in consistent condom use in anal intercourse. The positive effect was consistent in different study designs and by different measurement periods. However, HIV prevalence among MSM in China increased from 0.6% in 2003 to 7.4% in 2009 from a systematic review and meta-analysis [4]. The possible reasons for this contradiction might be: i) consistent condom use is a poor surrogate index of HIV risk because social desirability bias exists among MSM with consequent over-reporting of condom use; ii) prevention and control measures are not tailored to the needs and context of MSM communities, overestimating their effectiveness; iii) positive reports of program effectiveness might be easier to publish in peer-reviewed journals, resulting in a positive publication bias.

Unprotected anal intercourse (UAI) with casual sexual partners is known as an important route of HIV acquisition for MSM. Recent research has indicated that higher levels of UAI may be associated with one’s level of perceived familiarity with casual sexual partners [36]. Likewise, UAI with regular sexual partners has increasingly attracted attention in recent years [37], [38]. It is notable that behavioral interventions significantly increased consistent condom use during anal intercourse both with casual (41% increase) and regular sexual partners (52% increase) in our subgroup meta-analyses, involving 12 individual studies. Our stratified analyses by number of study sites, venue of recruiting participants, type of risk reduction interventions, sample size at baseline, and rigor score of study design also found significant increases of consistent condom use during anal sex in these specific subgroups. The evidence base currently provides general support for intervention approaches, and the efforts to better understand mechanisms of intervention effects and confirm positive effects in high rigor designs should be prioritized.

Client-initiated HIV testing and counseling, known as voluntary counseling and testing (VCT), and provider-initiated HIV testing and counseling in health facilities have helped millions of people learn their HIV status, but global coverage of HIV testing and counseling programs remains low, especially in China. Low levels of HIV prevalence and high levels of stigma and discrimination against people living with HIV/AIDS are disincentives for VCT [39], [40]. Many studies have confirmed intervention effects of VCT promotion [41], [42], [43], but this significant effect may wane over time since the intervention [41]. Eight of ten studies from China reported positive effects on seeking HIV testing, continuing 6- to 48-months after intervention. In our meta-analysis, a 55% increase in HIV test seeking was associated with the behavioral interventions, though one study from Hong Kong showed 29% decrease at 6 months after internet-based intervention [19]. Far more work is needed in China to identify the effectiveness of different interventions on various outcomes over time.

Correct HIV-related knowledge and positive attitudes toward HIV/AIDS have been used to evaluate the interventions among MSM, especially in China. Inconsistent scales for quantitative measurement of HIV-related knowledge and attitudes present substantial challenges for estimating overall effectiveness. Combining the individual studies using meta-analysis suggested a 77% increase of HIV-related knowledge involving 21 Chinese studies, and a 135% increase of HIV-related positive attitudes in three Chinese studies [20], [21], [22].

It is challenging and costly to measure incidence of HIV or other STD, particularly over a meaningful and substantial time period. To our knowledge, only one meta-analysis among two studies has been done and shown 80% reduction of STI acquisition (chlamydia or gonorrhea) among people living with HIV/AIDS [44], [45], [46]. Our study failed to observe reduction of HIV and syphilis infections among Chinese MSM by synthesizing the findings from ten serial cross-sectional studies that measured one or both infections, though various behavioral interventions were performed in some selected cities, though a 40% increase of consistent condom use was observed in the subgroup analysis of serial cross-sectional studies. The similar finding from a recent meta-analytic review showed that HIV prevalence among MSM has substantially increased from 2001–2009 across all Chinese regions [2]. More comprehensive behavioral and biomedical interventions are needed to control this ongoing disaster.

## Strengths and Limitations

The strength of our study is our thoroughness and methodological rigor of the meta-analysis for risk reduction interventions among Chinese MSM. Our elucidation of the impact of behavioral interventions on behavior and knowledge is useful in identifying ongoing research and service needs. Our analyses adjusted baseline data between study arms in evaluating the effect of interventions and combined continuous and categorical outcomes of targeted outcomes, something done rarely in other reviews.

Our meta-analysis has limitations as well. All studies used self-reported behaviors, knowledge and attitudes as the outcomes of interest, which might be subject to social desirability bias. Second, no comparison group was included in pre-and-post studies and non-randomization designs represented most of the included studies, contributing a large portion of heterogeneity and reducing the power of analysis. Third, major publication bias in the formal evaluations was found. Positive outcomes might be easier to be accepted by journals. Finally, although 12 databases were searched for the reviews and extensive check for completeness by cross-referencing were employed, we cannot exclude having missed a relevant study.

## Methods

### Literature Search and Study Selection

A literature search was conducted to identify studies evaluating the effectiveness of HIV risk reduction interventions among MSM in China. Twelve electronic databases were searched for publications in peer-reviewed journals through May 2013, including AMED (Allied and Complementary Medicine Database, Ovid Technologies, Inc., New York), British Nursing Index (Ovid Technologies, Inc., New York), CNKI (Tongfang Knowledge Network Technology Co., Ltd., Beijing, China), CQVIP (Chongqing VIP Information Co., Ltd., Chongqing, China), EMBASE (Elsevier, Amsterdam, The Netherlands), EconLit (The American Economic Association, New York), ERIC (Education Resources Information Centre, Institute of Education Sciences of the U.S. Department of Education, Washington), Ovid Medline (Ovid Technologies, Inc., New York), PsycINFO (American Psychological Association, Washington), Scopus (Elsevier, Amsterdam, The Netherlands), Wanfang Data (Chinese Ministry of Science & Technology, Beijing, China), and Web of Science (Thomson Scientific Technical Support, New York). The following combination of key words was used in literature search: (men who have sex with men OR MSM OR homosexual men OR gay men OR bisexual men OR transgender women OR money boy) AND (HIV OR AIDS OR sexually transmitted infections OR sexually transmitted diseases) AND (intervention OR randomized clinical trial OR treatment OR prevention OR adherence OR compliance). All publications were exported to an Endnote file (Endnote X4, Thomson Reuters, San Francisco, CA), and duplicates were deleted. The title and abstract of each paper were independently reviewed by two authors (Liu Y, and Dahiya K) to determine its relevance to the topic. Then, full texts were reviewed whether the paper assessed impacts of risk reduction intervention on HIV-related outcomes among MSM in China. Cross-referencing by checking the cited references in the included papers was also performed as an additional tool to identify relevant publications.

### Inclusion Criteria

Studies that met the following criteria were included in this meta-analysis: 1) studies evaluating the effectiveness of HIV risk reduction interventions among MSM, including randomized clinical trials (RCTs), quasi-experimental studies, pre-and-post intervention studies without control groups, and serial cross-sectional intervention studies; 2) studies conducted in China; 3) studies reporting HIV-related knowledge, attitudes and behaviors, as well as prevalence of HIV or other sexually transmitted infections (STIs); 4) published in English or Chinese. Duplication of human samples of included studies was evaluated by two authors and these samples were only used once in our analyses.

### Data Extraction

Data extraction was independently done by two authors (Liu Y, and Dahiya K) using a standardized form including items on lead author, publication year, study city, venue of recruiting participants, study design, demographic characteristics of study groups, characteristics of sex partners (regular or casual), description of intervention and comparison, duration of follow-up, drop-out rate, proportion or mean frequency of HIV-related outcomes at different follow-up time points, and rigor score of study design. Any disagreements between two data extractors were discussed with the team until a consensus was reached.

### Rigor Score

The rigor of study design for each study was assessed using an 8-item scale, as used in other reviews [47], [48] plus an additional item of sample size with a cut-off value of >100 representing good statistical power. The scale is additive, with 1 point for each item. Therefore, the rigor score ranges from 0 to 9, with a higher value representing better study design.

### Statistical Methods

We focused on six main outcomes in our meta-analysis: (1) consistent condom use, (2) uptake of HIV testing, (3) HIV-related knowledge, (4) HIV-related attitudes, (5) HIV infection, and (6) syphilis infection. For studies with multiple intervention arms [20], the effect sizes were calculated using the same comparison arm. When some studies had multiple measurements at different follow-up time points, the last follow-up assessment was used in the meta-analysis for estimating the overall effect size. When such outcome variables were not explicitly reported, they were derived from data provided in the paper or were secured from the authors when possible.

Effect size was calculated on the basis of targeted outcomes from the baseline and latest follow-up assessments between study arms (or self-pre-and-post intervention studies without control arms, or serial cross-sectional intervention studies). Standard mean differences (SMDs) and 95% confidence intervals (Cls) were used to estimate the effectiveness of risk reduction interventions. When studies reported dichotomous outcomes, we transformed odds ratios into SMDs using Cox transformation [49], [50]. SMD in each study arm was calculated as a fraction of dividing the difference of two means at follow-up and baseline by the pooled standard deviation (SD) of these two means [51]. The difference of SMDs from the intervention and comparison arms was used for meta-analysis. As the study arms might not be comparable at baseline, even in RCTs, we used Becker’s strategy to adjust for the reported difference between arms at baseline when calculating SMDs; for pre-and-post intervention studies without a comparison arm and for serial cross-sectional intervention studies, we assumed the value for the comparison arm was zero [51]. An SMD difference>0 indicated an increase in the given outcome in the intervention group relative to the control group. Random effects models were derived using the DerSimonian-Laird method [52], [53] to establish overall effect sizes. Random effects estimates allowed for variation of true effects across studies [54].

We assessed heterogeneities by I^2^ statistics [55], and identified outliers by standardized deleted residuals analyses. The funnel plot, Begg and Mazumdar rank correlation test, and Egger’s test of the intercept were employed to assess publication bias [56].

We conducted pre-planned subgroup analyses to examine consistent condom use during anal intercourse by type of sexual partners (regular vs. casual), length of recall period on consistent condom use (last sex vs. last 6 months), number of study site (one vs. multiple cities), venue of recruiting participants (establishment-based vs. other), type of risk reduction interventions (peer-led vs. other), study design (randomized clinical trial evaluation vs. quasi-experimental evaluation vs. self-pre-and-post intervention evaluation without control groups vs. serial cross-sectional intervention evaluation), sample size at baseline (≤300 vs. >300), and rigor score (1 vs. >1). We conducted sensitivity analyses to determine the stability of intervention effects by evaluating whether the overall effect size was sensitive to inclusion of each individual study. The R/S plus software version 2.15.1 was used for the meta-analyses [57].

## Conclusions

Our analysis suggested that available behavioral interventions can increase consistent condom use during anal sex, regardless of type of sexual partners, encourage successfully seeking of HIV testing, increase HIV-related knowledge and improve attitudes. But these interventions have had limited impacts on HIV or syphilis infection per se. Well-designed intervention studies are needed to explore the effectiveness of a variety of MSM-focused behavioral intervention programs in China.

## Supporting Information

Appendix S1 (DOCX)

Checklist S1 PRISMA 2009 Checklist.
(DOC)

## References

[1] Ministry of Health in People’s Republic of China, Joint United Nations Programme on HIV/AIDS, World Health Organization (2011) 2011 Estimates for the HIV/AIDS Epidemic in China. Beijing, China.

[2] Chow EP, Wilson DP, Zhang J, Jing J, Zhang L (2011) Human immunodeficiency virus prevalence is increasing among men who have sex with men in China: findings from a review and meta-analysis. Sex Transm Dis 38: 845–857. 21844741 10.1097/OLQ.0b013e31821a4f43

[3] Chow EP, Iu KI, Fu X, Wilson DP, Zhang L (2012) HIV and sexually transmissible infections among money boys in China: a data synthesis and meta-analysis. PLoS One 7: e48025. 23209551 10.1371/journal.pone.0048025 PMC3510224

[4] Meng X, Zou H, Beck J, Xu Y, Zhang X, et al (2013) Trends in HIV prevalence among men who have sex with men in China 2003–09: a systematic review and meta-analysis. Sex Health 10: 211–219. 23611402 10.1071/SH12093

[5] Wu Z, Xu J, Liu E, Mao Y, Xiao Y, et al.. (2013) HIV and Syphilis Prevalence Among Men Who Have Sex With Men: A Cross-Sectional Survey of 61 Cities in China. Clin Infect Dis: [Ahead of Print]. 10.1093/cid/cit210 PMC3689345 23580732

[6] Beyrer C, Baral SD, van Griensven F, Goodreau SM, Chariyalertsak S, et al (2012) Global epidemiology of HIV infection in men who have sex with men. Lancet 380: 367–377. 22819660 10.1016/S0140-6736(12)60821-6 PMC3805037

[7] Ye S, Xiao Y, Jin C, Cassell H, Blevins M, et al (2012) Effectiveness of integrated HIV prevention interventions among Chinese men who have sex with men: evaluation of a 16-city public health program. PLOS One 7: e50873. 23300528 10.1371/journal.pone.0050873 PMC3534092

[8] Yu J (2012) Teenage sexual attitudes and behaviour in China: a literature review. Health Soc Care Community 20: 561–582. 22404303 10.1111/j.1365-2524.2011.01054.x

[9] Gerbi GB, Habtemariam T, Tameru B, Nganwa D, Robnett V (2009) The correlation between alcohol consumption and risky sexual behaviors among people living with HIV/AIDS. J Subst Use 14: 90–100. 19693283 10.1080/14659890802624261 PMC2728293

[10] Shuper PA, Joharchi N, Irving H, Rehm J (2009) Alcohol as a correlate of unprotected sexual behavior among people living with HIV/AIDS: review and meta-analysis. AIDS Behav 13: 1021–1036. 19618261 10.1007/s10461-009-9589-z

[11] Gerbi GB, Habtemariam T, Tameru B, Nganwa D, Robnett V (2011) A comparative study of substance use before and after establishing HIV infection status among people living with HIV/AIDS. J Subst Use 16: 464–475. 22623879 10.3109/14659891.2010.495820 PMC3356919

[12] Huang Z, Wang M, Fu L, Fang Y, Hao J, et al (2013) Intervention to increase condom use and HIV testing among men who have sex with men in China: a meta-analysis. AIDS Res Hum Retroviruses 29: 441–448. 23083341 10.1089/AID.2012.0151

[13] Zheng L, Zheng Y (2012) Efficacy of human immunodeficiency virus prevention interventions among men who have sex with men in China: a meta-analysis. Sex Transm Dis 39: 886–893. 23064539 10.1097/OLQ.0b013e31826ae85e

[14] Hao C, Huan X, Yan H, Yang H, Guan W, et al (2012) A randomized controlled trial to evaluate the relative efficacy of enhanced versus standard voluntary counseling and testing on promoting condom use among men who have sex with men in China. AIDS Behav 16: 1138–1147. 22298340 10.1007/s10461-012-0141-1

[15] Tan JG, Cheng JQ, Lu ZX (2012) Evaluation of effects of combination intervention model to men who have sex with men. Zhonghua Yu Fang Yi Xue Za Zhi 46: 732–735. [Chinese]. 23157869

[16] Wang K, Yan H, Liu Y, Leng Z, Wang B, et al (2012) Increasing prevalence of HIV and syphilis but decreasing rate of self-reported unprotected anal intercourse among men who had sex with men in Harbin, China: results of five consecutive surveys from 2006 to 2010. Int J Epidemiol 41: 423–432. 22253304 10.1093/ije/dyr182

[17] Duan Y, Zhang H, Wang J, Wei S, Yu F, et al (2013) Community-based peer intervention to reduce HIV risk among men who have sex with men in Sichuan province, China. AIDS Educ Prev 25: 38–48. 23387950 10.1521/aeap.2013.25.1.38

[18] Guo W, Wu ZY, Song AJ, Poundstone K (2013) Impact of HIV/sexually transmitted infection testing on risky sexual behaviors among men who have sex with men in Langfang, China. Chin Med J (Engl) 126: 1257–1263. 23557555

[19] Lau JT, Lau M, Cheung A, Tsui HY (2008) A randomized controlled study to evaluate the efficacy of an Internet-based intervention in reducing HIV risk behaviors among men who have sex with men in Hong Kong. AIDS Care 20: 820–828. 18608057 10.1080/09540120701694048

[20] Gao Y, Wang S, Zhang S (2005) Success in adopting participatory approach to fight HIV through behavior change among gay men. Xian Dai Yu Fang Yi Xue 32: 1512–1515. [Chinese].

[21] Wang S, Gao Y, Zhang S (2005) Success in HIV-related health information diffusion and behaviour change among gay men, China. Zhong Guo Xing Bing Ai Zi Bing Yu Fang Kong Zhi Za Zhi 59: 109–112. [Chinese].

[22] Gao MY, Wang S (2007) Participatory communication and HIV/AIDS prevention in a Chinese marginalized (MSM) population. AIDS Care 19: 799–810. 17573601 10.1080/09540120601114832

[23] Zhang H, Zhu J, Wu Z, Pang L, Zhang L, et al.. (2009) Intervention trial on HIV/AIDS among men who have sex with men based on venues and peer network. Zhonghua Liu Xing Bing Xue Za Zhi 43: 970–976. [Chinese]. 20137518

[24] Song D, Tao XY, Cai WD, Wei AY, Huang GW (2005) Survey of sexual behavior of male homosexuals and intervention measure for control of AIDS in Shenzhen City. Zhong Guo Re Dai Yi Xue 5: 877–879. [Chinese].

[25] Zhu J, Zhang H, Wu Z, Zheng Y, Xu J, et al.. (2008) HIV risk behavior based on intervention among men who have sex with men peer groups in Anhui province. Zhonghua Yu Fang Yi Xue Za Zhi 42: 895–900. [Chinese]. 19141224

[26] He J, Dou Z, Wang F, Fang Y (2010) Estimate the effect of intervention models for control of AIDS among male homosexuals in Wuhu City. Shi Jie Gan Ran Za Zhi 10: 32–34. [Chinese].

[27] Meng XY, Shao Y (2010) Evaluation on the Effect of AIDS Related High-risk Behavior Intervention among MSM in Pulandian City. Prev Med Trib 16: 334–335. [Chinese].

[28] Wang YN, Liu JP, Xu QY, Zhang GP, Cai J, et al.. (2011) Effectiveness of health education on AIDS from non-governmental organizations among MSM. Xian Dai Yu Fang Yi Xue 38: 2523–2526.[Chinese].

[29] Xu J (2006) Assessment of effects of AIDS prevention & care project on men who have sex with men. Chin J AIDS STD 12: 215, 231-212. [Chinese].

[30] Zhang Z, Wen X, Chen W, Zhou Y (2010) Evaluation of intervention on AIDS related high risk behaviors among men having sex with men in Guilin. Yu Fang Yi Xue Qing Bao Za Zhi 26: 784–787. [Chinese].

[31] Wu GF, He FM, Heng SY (2011) Intervention effects evaluation on AIDS-related knowledge and high-risk Behaviors among 244 MSM in colleges. Anhui J Prev Med 17: 86–88. [Chinese].

[32] Xu J, Zhou W, Zhou DJ, Yao ZZ, Ding J, et al.. (2009) Evaluation of integrative behavioral intervention on AIDS among MSM in Wuhan. Chin J Health Edu 25: 736–738. [Chinese].

[33] Wang Y, Zhang HB, Zhang GG, Yang HW, Fan J (2008) Effectiveness of AIDS related health education and behavioral intervention in Mianyang MSM group. J Prev Med Inf 24: 962–967. [Chinese].

[34] Ding XB, Feng LG, Xiao Y, Jin CR, Xu SM, et al.. (2010) Treatment efficacy of behavior intervention on male homosexual AIDS patients. J Tropical Med 10: 323–326. [Chinese].

[35] Li JJ, Huan XP, Yan HJ, Zhang M, Tang WM, et al.. (2011) Effectiveness of behavioral intervention combined with voluntary counseling and testing on high risk behaviors among MSM. Chin Prev Med 12: 666–669. [Chinese].

[36] van den Boom W, Stolte I, Sandfort T, Davidovich U (2012) Serosorting and sexual risk behaviour according to different casual partnership types among MSM: the study of one-night stands and sex buddies. AIDS Care 24: 167–173. 21861633 10.1080/09540121.2011.603285 PMC3561689

[37] Slavin S, Richters J, Kippax S (2004) Understanding of Risk Among HIV Seroconverters in Sydney. Health Risk Society 6: 39–52.

[38] Blais M (2006) Vulnerability to HIV among regular male partners and the social coding of intimacy in modern societies. Cult Health Sex 8: 31–44. 16500823 10.1080/13691050500391232

[39] World Health Organization, UNAIDS, UNICEF (2011) Global HIV/AIDS response: epidemic update and health sector progress towards universal access: progress report 2011.

[40] International HIV/AIDS Alliance Asia and Eastern Europe team (April 2004) Voluntary Counselling and Testing. Emerging approaches from Asia and Eastern Europe.

[41] Vidanapathirana J, Abramson MJ, Forbes A, Fairley C (2005) Mass media interventions for promoting HIV testing. Cochrane Database Syst Rev: CD004775. 10.1002/14651858.CD004775.pub2 PMC13429287 16034948

[42] Davis KC, Uhrig J, Rupert D, Fraze J, Goetz J, et al (2011) Effectiveness of a mass media campaign in promoting HIV testing information seeking among African American women. J Health Commun 16: 1024–1039. 21707409 10.1080/10810730.2011.571342

[43] Sweat M, Morin S, Celentano D, Mulawa M, Singh B, et al (2011) Community-based intervention to increase HIV testing and case detection in people aged 16–32 years in Tanzania, Zimbabwe, and Thailand (NIMH Project Accept, HPTN 043): a randomised study. Lancet Infect Dis 11: 525–532. 21546309 10.1016/S1473-3099(11)70060-3 PMC3156626

[44] Wingood GM, DiClemente RJ, Mikhail I, Lang DL, McCree DH, et al.. (2004) A randomized controlled trial to reduce HIV transmission risk behaviors and sexually transmitted diseases among women living with HIV: The WiLLOW Program. J Acquir Immune Defic Syndr 37 S58–67. 10.1097/01.qai.0000140603.57478.a9 15385901

[45] Wolitski RJ, Gomez CA, Parsons JT (2005) Effects of a peer-led behavioral intervention to reduce HIV transmission and promote serostatus disclosure among HIV-seropositive gay and bisexual men. AIDS 19 S99–109. 10.1097/01.aids.0000167356.94664.59 15838199

[46] Crepaz N, Lyles CM, Wolitski RJ, Passin WF, Rama SM, et al (2006) Do prevention interventions reduce HIV risk behaviours among people living with HIV? A meta-analytic review of controlled trials. AIDS 20: 143–157. 16511407 10.1097/01.aids.0000196166.48518.a0

[47] Medley A, Kennedy C, O’Reilly K, Sweat M (2009) Effectiveness of peer education interventions for HIV prevention in developing countries: a systematic review and meta-analysis. AIDS Educ Prev 21: 181–206. 19519235 10.1521/aeap.2009.21.3.181 PMC3927325

[48] Kennedy CE, Medley AM, Sweat MD, O’Reilly KR (2010) Behavioural interventions for HIV positive prevention in developing countries: a systematic review and meta-analysis. Bull World Health Organ 88: 615–623. 20680127 10.2471/BLT.09.068213 PMC2908966

[49] Cox DR (1970) Analysis of binary data. New York: Chapman & Hall/CRC.

[50] Sanchez-Meca J, Marin-Martinez F, Chacon-Moscoso S (2003) Effect-size indices for dichotomized outcomes in meta-analysis. Psychol Methods 8: 448–467. 14664682 10.1037/1082-989X.8.4.448

[51] Becker BJ (1988) Synthesizing standardized mean-change measures. Br J Math Stat Psychol 41: 257–278.

[52] DerSimonian R, Laird N (1986) Meta-analysis in clinical trials. Control Clin Trials 7: 177–188. 3802833 10.1016/0197-2456(86)90046-2

[53] Lipsey M, Wilson D (2001) Practical meta-analysis: Thousand Oaks, CA: Sage.

[54] Normand SL (1999) Meta-analysis: formulating, evaluating, combining, and reporting. Stat Med 18: 321–359. 10070677 10.1002/(sici)1097-0258(19990215)18:3<321::aid-sim28>3.0.co;2-p

[55] Deeks J, Altman D, Bradburn M (2002) Statistical methods for examining heterogeneity and combining results from several studies in a meta-analysis. In: Egger M, Davey Smith G, Altman D, editors. Systematic reviews in health care: meta-analysis in context. London: London: BMJ Publications. 285–312 p.

[56] Rothstein HR, Sutton AJ, Borenstein M (2005) Publication Bias in Meta-Analysis: Prevention, Assessment and Adjustments: Chichester, England: Wiley.

[57] R/S plus Software can be downloaded free. Available: http://127.0.0.1:13917/doc/html/index.html.

[58] Liu F, Liu HH, Zhou C, Liu L, Yi D (2008) Effect of integrated intervention on HIV/AIDS of MSM in a certain District of Chongqing. Pract Prev Med 15: 1055–1058. [Chinese].

[59] Cao NX, Jiang J, Ge FQ, Li Q, Wang XD, et al.. (2009) Effectiveness of comprehensive interventions for STD/HIV/AIDS prevention amongmale sexworkers. Chin J AIDS STD 15: 497–482. [Chinese].

[60] Feng L, Ding X, Lv F, Pan C, Yi H, et al.. (2009) Study on the effect of intervention about acquired immunodeficiency syndrome among men who have sex with men. Zhonghua Liu Xing Bing Xue Za Zhi 30: 18–20. [Chinese]. 19565841

[61] Wang M, Wu CX (2009) Cognitive status of AIDS and the effects of health intervention among YMSM in Wuhan city. J Pub Health Prev Med 20: 43–46. [Chinese].

[62] Wang Y, Zhang HB, Li ZJ, Xu J, Zhang GG, et al.. (2009) Effect evaluation on behavior intervention of AIDS/STI prevention and treatment in MSM group. Pract Prev Med 16: 654–657. [Chinese].

[63] Xiu CZ, Liu MH, Li XF, Fa P, Jiang ZX, et al.. (2009) Study of AIDS knowledge, behavior, intervention and the status of infection among MSM. Prev Med Trib 15: 97–100,103. [Chinese].

[64] Zeng G, Xiao Y, Xu P, Feng N, Jin C, et al.. (2009) Evaluation of effect of community-based HIV/AIDS interventions among men who have sex with men in eighteen cities, China. Zhonghua Liu Xing Bing Xue Za Zhi 43: 977–980. [Chinese]. 20137519

[65] Ma GL, Shen LT, Su C-hZH-nLB-yLL (2010) Effect evaluation of HIV/AIDS integrated intervention of MSM in Xiamen City. Chin J Dis Control Prev 14: 726–728. [Chinese].

[66] Nong QX, Xu YF, Lin XQ, Chen SH, Zhu JJ, et al.. (2010) Evaluation on HIV/AIDS intervention among men having sex with men. J Prev Med Inf 26: 200–203. [Chinese].

[67] Qu L, Tao B, JQ D, Yang JY, Bao ZQ (2011) Evaluation on the comprehensive acquired immunodeficiency syndrome intervention program conducting in men who have sex with men population in Inner Mongolia. Chin J Infect Dis 29.

[68] Wang FX, Huang YL (2011) Analysis on effect of AIDS related high risk behavior intervention among MSM in Yingtan City, 2009–2010. Prev Med Trib 17: 518–520, 524. [Chinese].

[69] Wang L, Liu ZF, Cao WH (2011) Effect evaluation of testing initiated HIV/AIDS comprehensive interventions among men who have sex with men. Chin Prim Health Care 25: 94–95. [Chinese].

