# Supplementary material for: Effectiveness of HIV Risk Reduction Interventions among Men who have Sex with Men in China: A Systematic Review and Meta-Analysis
Source: PLoS One. 2013 Aug 30;8(8):e72747. doi: 10.1371/journal.pone.0072747 (PMC3796948; doi:10.1371/journal.pone.0072747)
Supplement: Appendix S1 — (DOCX) [file pone.0072747.s001.docx]

**Supplementary material S1:** The list of studies excluded from this review because of:

*(A)* *Not original articles but editorials, comments, reviews*

1. Chow EP, Wilson DP, Zhang J, Jing J, Zhang L (2011) Human immunodeficiency virus prevalence is increasing among men who have sex with men in China: findings from a review and meta-analysis. Sex Transm Dis 38: 845-857.
2. Huang Z, Wang M, Fu L, Fang Y, Hao J, et al. (2013) Intervention to increase condom use and HIV testing among men who have sex with men in China: a meta-analysis. AIDS Res Hum Retroviruses 29: 441-448.
3. Li J, Chen XS (2012) Application of Internet as a tool for HIV/STD behavioral survey and intervention among MSM. Chin J AIDS STD 18: 62-64. [Chinese].
4. Meng X, Zou H, Beck J, Xu Y, Zhang X, et al. (2013) Trends in HIV prevalence among men who have sex with men in China 2003?09: a systematic review and meta-analysis. Sex Health.
5. van Griensven F, de Lind van Wijngaarden JW (2010) A review of the epidemiology of HIV infection and prevention responses among MSM in Asia. AIDS 24 S30-40.
6. Wei C, Herrick A, Raymond HF, Anglemyer A, Gerbase A, et al. (2011) Social marketing interventions to increase HIV/STI testing uptake among men who have sex with men and male-to-female transgender women. Cochrane Database Syst Rev: CD009337.
7. Yun K, Xu JJ, Reilly KH, Zhang J, Jiang YJ, et al. (2011) Prevalence of bisexual behaviour among bridge population of men who have sex with men in China: a meta-analysis of observational studies. Sex Transm Infect 87: 563-570.
8. Zheng L, Zheng Y (2012) Efficacy of human immunodeficiency virus prevention interventions among men who have sex with men in China: a meta-analysis. Sex Transm Dis 39: 886-893.

*(B) Not intervention specified*

1. Thomas B, Mimiaga MJ, Mayer KH, Johnson CV, Menon S, et al. (2009) HIV prevention interventions in Chennai, India: are men who have sex with men being reached? AIDS Patient Care STDS 23: 981-986.
2. Thomas B, Mimiaga MJ, Menon S, Chandrasekaran V, Murugesan P, et al. (2009) Unseen and unheard: predictors of sexual risk behavior and HIV infection among men who have sex with men in Chennai, India. AIDS Educ Prev 21: 372-383.
3. van Griensven F, Thienkrua W, Sukwicha W, Wimonsate W, Chaikummao S, et al. (2010) Sex frequency and sex planning among men who have sex with men in Bangkok, Thailand: implications for pre- and post-exposure prophylaxis against HIV infection. J Int AIDS Soc 13: 13.
4. Zou H, Wu Z, Yu J, Li M, Ablimit M, et al. (2013) Internet-facilitated, voluntary counseling and testing (VCT) clinic-based HIV testing among men who have sex with men in China. PLOS One 8: e51919.

*(C) Lack of information on target outcomes or measures of interest*

1. Chandrashekar S, Guinness L, Kumaranayake L, Reddy B, Govindraj Y, et al. (2010) The effects of scale on the costs of targeted HIV prevention interventions among female and male sex workers, men who have sex with men and transgenders in India. Sex Transm Infect 86 i89-94.
2. Dandona L, Kumar SG, Kumar GA, Dandona R (2009) Economic analysis of HIV prevention interventions in Andhra Pradesh state of India to inform resource allocation. AIDS 23: 233-242.
3. Wu C, Yu Q, Zhu C, Xu S, Dai H (2006) Influence of the companion education on the change of AIDS knowledge and attitudes among the MSM. J Public Health Prev Med 17: 16-19. [Chinese].

*(D) Repeated report from the same study*

1. Zhang H, Wu Z, Zheng Y, Wang J, Zhu J, et al. (2010) A pilot intervention to increase condom use and HIV testing and counseling among men who have sex with men in Anhui, China. J Acquir Immune Defic Syndr 53: S88-92.
